# The color of greater flamingo feathers fades when no cosmetics are applied

**DOI:** 10.1002/ece3.8041

**Published:** 2021-09-23

**Authors:** Maria Cecilia Chiale, Miguel A. Rendón, Sophie Labaude, Anne‐Sophie Deville, Juan Garrido‐Fernández, Antonio Pérez‐Gálvez, Araceli Garrido, Manuel Rendón‐Martos, Arnaud Béchet, Juan A. Amat

**Affiliations:** ^1^ Laboratorio de Histología y Embriología Descriptiva Experimental y Comparada Facultad de Ciencias Veterinarias Universidad Nacional de La Plata La Plata Argentina; ^2^ CONICET La Plata Argentina; ^3^ Departamento de Ecología de Humedales Estación Biológica de Doñana, C.S.I.C. Sevilla Spain; ^4^ Tour du Valat Institut de Recherche pour la Conservation des Zones Humides Méditerranéennes Le Sambuc Arles France; ^5^ Departamento de Fitoquímica de Alimentos Instituto de la Grasa, C.S.I.C. Sevilla Spain; ^6^ Agencia de Medio Ambiente y Agua de Andalucía Consejería de Agricultura, Ganadería, Pesca y Desarrollo Sostenible Málaga Spain; ^7^ Reserva Natural Laguna de Fuente de Piedra Consejería de Agricultura, Ganadería, Pesca y Desarrollo Sostenible Fuente de Piedra Spain

**Keywords:** carotenoids, makeup, plumage coloration, signaling, uropygial secretions

## Abstract

Greater flamingos use cosmetic coloration by spreading uropygial secretions pigmented with carotenoids over their feathers, which makes the plumage redder. Because flamingos inhabit open environments that receive direct solar radiation during daytime, and carotenoids bleach when exposed to solar radiation, we expected that the plumage color would fade if there is no maintenance for cosmetic purposes. Here, we show that the concentrations of pigments inside feathers and on the surface of feathers were correlated, as well as that there was a correlation between the concentrations of pigments in the uropygial secretions and on the surface of feathers. There was fading in color (becoming less red) in feathers that received direct solar radiation when there was no plumage maintenance, but not so in others maintained in darkness. When we controlled for the initial color of feathers, the feathers of those individuals with higher concentration of pigments on the feather surfaces were those that lost less coloration after experimental exposure of feathers to sunny conditions. These results indicate that exposure to sunlight is correlated with the fading of feather color, which suggests that individuals need to regularly apply makeup to be more colorful. These results also reinforce the view that these birds use cosmetic coloration as a signal amplifier of plumage color. This may be important in species using highly variable habitats, such as wetlands, since the conditions experienced when molting may differ from those when the signal should be functional, usually months after molting.

## Abstract 2

Greater flamingo plumage coloration fades when there is no frequent application of cosmetics.

## 1 INTRODUCTION

The color of feathers is due to pigmentation or structural coloration (Gill, 1990). Because feathers are inert tissue and pigments are deposited into feathers during molt (Gill, 1990), it has for long been assumed that feather coloration was a relatively static trait. But there are an increasing number of evidences showing that feather coloration may change between molting periods, even during short‐term ones. This may be due to accidental staining with organic or inorganic substances (Ficken & Ficken, 1962; Kennard, 1918; Shawkey et al., 2011; Surmacki, 2011; Surmacki & Nowakowski, 2007), abrasion (Fig uerola & Senar, 2004; Surmacki et al., 2011; Veiga, 1996; Willoughby et al., 2002), saprophytic fungal growth (Clubb & Herron, 1998), denaturation of pigments (Blanco et al., 2005), or to the deliberate application of some substances over the feathers by the birds themselves (i.e., cosmetic coloration; Delhey et al., 2007; Montgomerie, 2006; Negro et al., 1999; van Overheld et al., 2017; Pérez‐Rodríguez et al., 2011). Both male and female greater flamingos *Phoenicopterus roseus* apply over their plumage, as makeup, uropygial secretions pigmented with carotenoids that make their feathers redder (Amat et al., 2011, 2018). In this species, the redder individuals breed earlier and are preferred as mates (Amat et al., 2011; Freeman et al., 2016), and it has been suggested that plumage color would act as a dynamic signal of parental quality (Amat et al., 2018; Amat & Rendón, 2017).

Up to six types of carotenoids are present in the plumage and other tissues of flamingos (Amat & Rendón, 2017). Of such pigments, canthaxanthin is the main carotenoid deposited in greater flamingo feathers (>60% of all carotenoids, Fox et al., 1967; Amat et al., 2011), which is also accumulated in the uropygial secretions that these birds use as makeup (Amat et al., 2011). Carotenoid pigments bleach when exposed to environmental conditions (Christophersen et al., 1991; Mortensen & Skibsted, 1999; Surmacki et al., 2011; Töpfer, 2012). The bleaching of plumage may be especially acute in birds that use substances pigmented with carotenoids as makeup and especially if they inhabit open environments receiving direct solar radiation throughout the day, such as flamingos, because the degradation of the pigments external to the plumage would be rather rapid. This is expected because UV‐visible radiation is the main contributor to carotenoid degradation (Örnborg et al., 2002) and would explain why flamingo feathers rapidly lose their coloration once shed (Kight, 2015). Therefore, a frequent application of cosmetic uropygial secretions over feathers would be necessary to maintain the plumage colorful. Such a possibility encouraged us to experimentally test whether the plumage color of greater flamingos fades when no uropygial secretions are applied over feathers with cosmetic purposes and the feathers remain exposed to sunlight. We predicted that the degree of plumage fading when there is no plumage maintenance should be related to the quantity of pigments deposited as makeup over feathers’ surfaces.

## 2 MATERIALS AND METHODS

**FIGURE 1:**
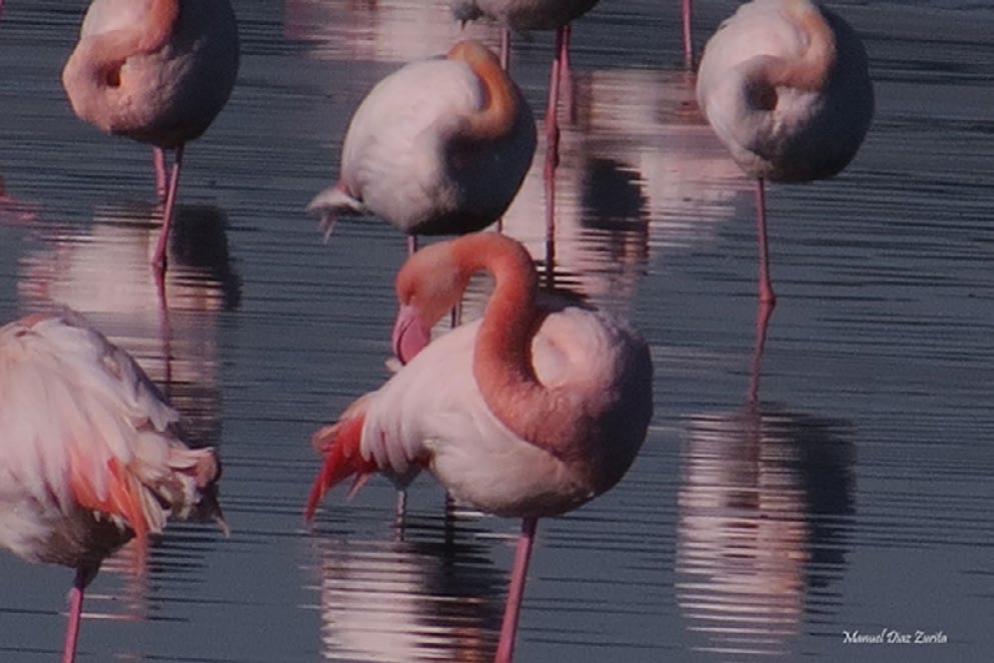
The red coloration in neck and head feathers of greater flamingos results from the application by the birds themselves of uropygial secretions pigmented with carotenoids (photograph credit: Manuel Díaz Zurita)

We used for this study 30 adult greater flamingos found recently dead during a cold spell in the Camargue, southern France, in February 2012 (Deville et al., 2014). From every bird, we plucked together 5–10 feathers from the neck, since greater flamingos use cosmetic coloration on these feathers (Amat et al., 2011, 2018) (Figure 1). We also excised the uropygial gland from every bird. All samples were kept at −80℃ until pigment analyses and measurement of color.

### 2.1 Carotenoids in feathers

We used a set of feathers (one feather per bird) to obtain carotenoids on their surface (i.e., external to feathers), following the procedure described in Amat et al. (2011). Briefly, the feathers were individually weighed to the nearest 0.1 mg using a Mettler Toledo electronic balance and introduced in 15‐ml Falcon tubes filled with acetone for pigment extraction. These tubes were shaken during 2 min, using Vortex, after which they were subject to sonication during 2 min. Pigment extracts were transferred to a rotatory flask, and the solvent was evaporated until it was dry. The residue was dissolved in 100–300 μl of N,N‐dimethylformamide (the quantity varied depending on the intensity of the apparent color), filtered through a nylon net (0.45‐μm mesh size) into an Eppendorf tube, and stored at −30℃ until analysis, which was performed within 1 week after extraction. This procedure did not remove any pigment internal to feathers (Amat et al., 2011).

Subsequently, we extracted carotenoids internal to feathers from the same set of feathers, for which we employed a protocol similar to that of Blanco et al. (2005). The individual neck feathers from which the external carotenoids had been previously removed were placed within folded filter paper and introduced into 15‐ml Falcon tubes for subsequent extraction of pigments. Ten milliliters of N, N‐dimethylformamide was added, and the tube was placed at 60℃ for 60 min, including sonication for 5 min every 30 min. The extraction procedure was repeated three times with acetone as extraction solvent. All fractions were pooled in a separator funnel and treated with 50 ml of diethyl ether to collect the pigments. A sufficient amount of 10% NaCl was added to allow the separation of the phases. The ether phase was filtrated through anhydrous sodium sulfate, evaporated in a rotary evaporator, and taken up to 0.2 ml with acetone. Resulting extracts were subsequently centrifuged at 12,000 *g* for 5 min and the upper layer stored at −30℃ until analyzed.

Extraction of carotenoids was done using high‐performance liquid chromatography (HPLC). Identification of carotenoids was performed by comparison of the chromatographic behavior of the peaks (retention time and UV‐visible spectra) with that of authentic standards, while quantification was performed using external calibration curves from injection of progressive concentrations of the reference pigment (see full details in Amat et al., 2011). The content of canthaxanthin in feathers obtained from each sample was expressed as mg of canthaxanthin/g of feather.

### 2.2 Carotenoids in uropygial secretions

In the laboratory, we collected up to 50 mg of uropygial secretions per sample from the uropygial glands and transferred them into 2‐ml Eppendorf tubes for subsequent extraction of pigments. We recorded to the nearest 0.1 mg the mass of secretions of every sample. For carotenoid extraction, we added 60–150 μl of acetone (the quantity varied depending on the intensity of the apparent color) to each sample of uropygial secretions. The mixture was shaken for 2 min, and subsequently, it was subject to sonication for 2 min. The sample was then centrifuged at 12,000 *g* for 5 min and the upper layer stored at −30℃ until analysis using HPLC. The content of canthaxanthin in the uropygial secretions was expressed as mg of canthaxanthin/g of secretion.

### 2.3 Color changes in feathers

To study whether the color fades when there is no plumage maintenance by the birds, we attached two groups of neck feathers of individual flamingos (*n* = 30) by the calamus with transparent adhesive tape on sheets of black cardboard (Blanco et al., 2005; Hasegawa et al., 2008; Pearlstein & Keene, 2010). The sheets were inserted into transparent plastic bags to avoid being covered by dust. These sets of feathers were different from that in which carotenoids were measured, and were exposed to ambient outdoor conditions for 40 consecutive days on a flat roof. The treatment group received direct solar radiation. The control group of feathers did not receive solar radiation, as the side of the sheet on which the feathers were attached was facing down. We scanned individual feathers using an Epson Perfection 1250 scanner, just before exposing them to ambient conditions (day 0), and again after exposition to ambient conditions (day 40). Scanned images of feathers were saved as JPEG files, from which coloration was determined in the Commission International d’Eclairage (CIE) L*a*b* color space, in which L* represents lightness, a* the red/green value (redness), and b* the yellow/blue value (yellowness). We obtained a* values using Adobe Photoshop CS4 (Adobe, San José, California, USA), for which the eyedropper was set at 5 × 5 pixels and placed over five points along the feather images (for details, see Amat et al., 2011). We quantified color in this way instead of considering the avian color space because we were interested in estimating changes in redness, and this is appropriate for our purpose, since greater flamingos are able of perceiving changes in the intensity of red coloration (Amat et al., 2018). The visual system of the greater flamingo is categorized as violet‐sensitive (Ödeen & Håstad, 2003), and we did not consider reflectance in this part of the spectrum, though it may be important in avian signaling (e.g., Eaton & Lanyon, 2003).

### 2.4 Statistical analyses

We used STATISTICA (Dell, 2015) to perform statistical tests. We checked the data for normality and homoscedasticity, and when these criteria were not met, we used nonparametric statistical tests instead of parametric ones. For each feather and measurement day, we averaged the five values of a*. We compared the concentrations of canthaxanthin inside the feathers and on the surface of feathers using Spearman rank correlation (*r_s_*). This last test was also used to compare the concentrations of canthaxanthin in the uropygial secretions and on the surface of feathers. We used Pearson´s correlation (r) to check whether there was a relationship between the initial a* values (a*_0_) in both groups of feathers (i.e., those receiving direct solar radiation vs. those in darkness). We used generalized linear mixed models (GLMM) to compare initial (day 0) and final (day 40) a* values each one of the groups of samples, considering individuals as a random factor, period (initial and final measurements, i.e., day 0 and day 40, respectively) as categorical factor, and a* values as dependent variable.

We estimated the degree of color change for each feather after 40 days of exposition relative to initial values, as: ([a*_0_ − a*_40_]/a*_0_). We used the average a*_0_ values for the calculation of the relative changes in color. We compared the concentration of canthaxanthin external to feathers (i.e., the pigments used as cosmetics) with the change in feather color after 40 days of exposition to sunny conditions, while controlling for initial color values (a*_0_), using Kendall partial rank‐order correlation (Siegel & Castellan, 1988). Although the concentrations of pigments and the changes in coloration were actually measured in different feathers of the same individuals, for this partial correlation we assumed that in every individual bird all neck feathers received very similar amounts of uropygial secretions over them. Indeed, from each flamingo, we plucked the feathers together, that is, one feather was in immediate proximity to the others.

## 3 RESULTS

The average concentrations of canthaxanthin (±*SD*) were as follows: 0.14 ± 0.26 mg/g on the surface of feathers, 9.20 ± 6.92 mg/g inside the feathers, and 0.53 ± 0.44 mg/g in the uropygial secretions (*n* = 30 in all cases). There was a significant correlation between the concentrations of canthaxanthin inside the feathers and on the surface of feathers (*r_s_* = 0.49, *n* = 30, *p* < 0.01). There was also a significant correlation between the concentrations of canthaxanthin in the uropygial secretions and on the surface of feathers (*r_s_* = 0.40, *n* = 30, *p* < 0.025).

**FIGURE 2:**
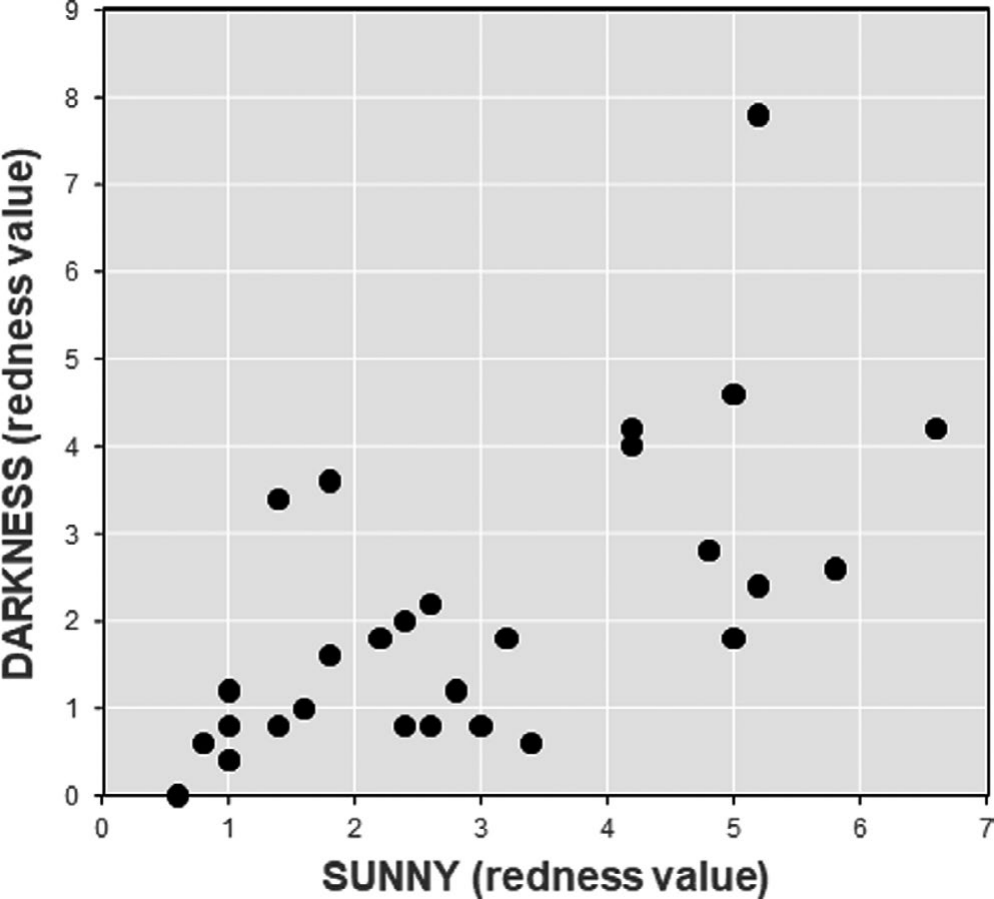
Relationship between the redness of neck feathers from greater flamingos before the exposure of feathers to sunny conditions and the maintenance of feathers under conditions of darkness (*n* = 30 in each group). The redness values were obtained from the Commission International d'Eclairage (CIE) L*a*b* color space

**FIGURE 3:**
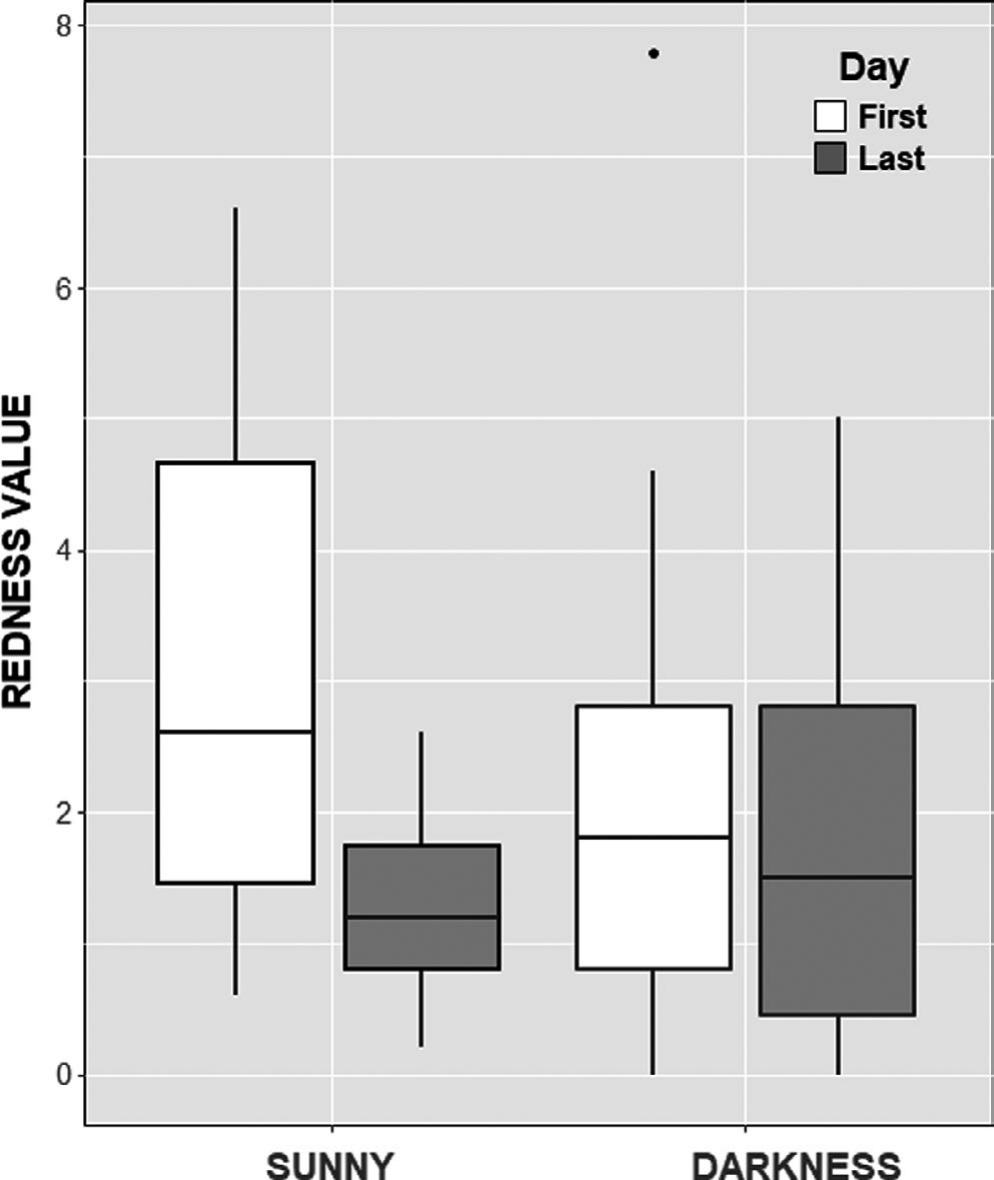
Boxplots for redness values of neck feathers from greater flamingos. The feathers either received direct solar radiation or were in darkness (*n* = 30 in each group) during 40 days. The boxes represent the interquartile ranges (IQR: 25th and 75th percentiles), and the lines within the boxes are the medians. Whiskers extend 1.5 × IQR from the 75th and 25th percentiles. A dot denotes an outlier. The redness values were obtained from the Commission International d’Eclairage (CIE) L*a*b* color space

There was a highly significant correlation between the a*_0_ values of feathers that were exposed to sunny conditions and those of feathers maintained in darkness before exposition of feathers to environmental conditions (*r*_28_ = 0.65, *p* = 0.001; Figure 2). The feathers exposed to direct solar radiation were significantly duller after 40 days of exposition, with a fading in redness of 57.8% relative to initial values (GLMM, *F*_1,29_ = 46.3, *p* < 0.001; Figure 3). However, there were slight, marginally not significant, changes in the redness of feathers after 40 days of exposition to ambient conditions when the feathers did not receive solar radiation, with a fading in redness of 11.4% relative to initial values (GLMM, *F*_1,29_ = 3.9, *p* = 0.058; Figure 3). When we controlled for the initial color of feathers (a*_0_), the feathers of those individuals with higher concentration of canthaxanthin on their surfaces were those that lost less coloration after exposition to solar radiation (Kendall partial correlation, *T* = −0.37, *n* = 30, *p* < 0.005).

## 4 DISCUSSION

Our results show that there was an evident fading in coloration (becoming less red) in neck feathers of greater flamingos that received direct solar radiation after 40 days in which there was no plumage maintenance by the birds. Although we found a slight fading in the coloration of flamingo feathers maintained in darkness, the change in coloration was not as apparent as in feathers maintained under sunny conditions, which may be linked to the fact that UV‐visible radiation is the main factor causing carotenoid degradation (Örnborg et al., 2002).

Furthermore, feathers with higher amounts of carotenoids on their surface lost less coloration, which suggests a protective role of the thickness of the carotenoid layer. Following chick hatching, the adult greater flamingos use much less frequently the behavior employed to spread the uropygial secretions over their plumage with cosmetic purposes than during the months preceding chick hatching, which together with a decrease in the concentration of carotenoids in the uropygial secretions results in a fading of plumage coloration in about a month (Amat et al., 2011, 2018). In these studies, it was suggested that to maintain the plumage colorful, flamingos would need to apply frequently makeup over their feathers during the long displaying period, which in the Mediterranean region spans October–May (Amat et al., 2011; Johnson & Cézilly, 2007). Here, we demonstrate that the neck plumage coloration would fade during such a long period when there is no possibility of plumage maintenance. This result is consistent with the finding that the more colorful individuals apply cosmetics over their feathers more frequently than the less colorful individuals (Amat et al., 2011), which suggests that colorful individuals need to regularly apply makeup to be more colorful.

The flamingos used in this study might have died of starvation, and it is possible that in these individuals the assignment of carotenoids to the uropygial gland for cosmetic purposes was not as frequent as in healthy individuals, probably because starving individuals might allocate the carotenoids to another function (Morehouse, 2014). However, the cold spell happened suddenly, when flamingos where actively displaying (before starving; Labaude, Deville, & Béchet, pers. observ.), so that they had presumably already sprayed uropygial secretions on their feathers. Indeed, our study shows that neck feathers had pigments over their surface, presumably deposited with the uropygial secretions, and that the coloration of feathers fades when exposed to sunlight conditions if there is no plumage maintenance with cosmetic purposes.

It might be that the changes in color of flamingo feathers were not only due to bleaching of carotenoids external to feathers, but also to bleaching of carotenoids internal to feathers, as it has been shown in the great tit *Parus major* and the blue‐tailed bee‐eater *Merops philippinus* (Surmacki, 2008; Surmacki, Siefferman, et al., 2011). However, the plumages of the tit and the bee‐eater are pigmented with xanthophylls, and in these carotenoids, the oxidative degradation, causing bleaching, is more rapid than that of canthaxanthin (Woodall et al., 1997). The scarlet ibis *Eudocimus ruber*, whose plumage is mainly pigmented with canthaxanthin (as flamingos) (Fox, 1962), apparently does not use uropygial secretions with cosmetic purposes (Amat et al., unpubl.). However, in this species there are no colorimetric changes under visible light after short‐term (29 days) exposition of feathers to sunny conditions, when there is a lack of plumage maintenance (Pearlstein et al., 2014). What the results of Pearlstein et al. (2014) suggest is that when there are no canthaxanthin pigments external to feathers, there are no apparent changes in feather coloration in the visible part of the spectrum after a few weeks of feathers exposure to sunny conditions, though there may be changes in the UV part of the spectrum (Pearlstein et al., 2014). Therefore, the changes in redness that we found in flamingo feathers would be mainly due to bleaching of carotenoids external to feathers when there is a lack of maintenance for cosmetic purposes. In line with this, our results indicate that the degree of fading of plumage coloration in the greater flamingo is negatively correlated with the concentration of pigments over the feathers’ surfaces, which demonstrates the importance of the use of cosmetics for the maintenance of colorful plumage.

A previous study found that the intensity of plumage coloration in flamingos increased with the quantity of pigments used as cosmetics and suggested that the use of cosmetics is a signal amplifier of plumage color (Amat et al., 2011). The results of our study support this, as there is a relationship between the concentrations of canthaxanthin inside the feathers and on the surface of feathers. This would make differences in coloration easier to assess by the receivers of the signal (Hasson, 1991) because the use of cosmetics would bring out the coloration. This may be especially important in species inhabiting highly variable environments and that mate long before breeding, like flamingos. Indeed, in the Mediterranean this species generally molts at the end of the summer after the breeding season, while courtship displays extend from October to May. Hence, the conditions at times when the signal should be functional may differ from those when the color of plumage was acquired during molt so that the use of cosmetic coloration may provide a better image of the individual quality than the one indicated by the color acquired when molting several months before (Botero & Rubenstein, 2012; Montgomerie, 2006; Searcy & Nowicki, 2005).

## CONFLICT OF INTEREST

The authors declare that they have no competing interests.

## AUTHOR CONTRIBUTIONS

**Maria Cecilia Chiale:** Formal analysis (equal); Software (equal); Validation (equal); Visualization (equal); Writing‐original draft (equal); Writing‐review & editing (equal). **Miguel A. Rendón:** Formal analysis (equal); Software (equal); Validation (equal); Visualization (equal); Writing‐review & editing (equal). **Sophie Labaude:** Methodology (equal); Visualization (equal); Writing‐review & editing (equal). **Anne‐Sophie Deville:** Methodology (equal); Visualization (equal); Writing‐review & editing (equal). **Juan Garrido‐Fernández:** Data curation (equal); Funding acquisition (equal); Investigation (equal); Methodology (equal); Resources (equal); Visualization (equal); Writing‐review & editing (equal). **Antonio Pérez‐Gálvez:** Data curation (equal); Funding acquisition (equal); Investigation (equal); Methodology (equal); Resources (equal); Visualization (equal); Writing‐review & editing (equal). **Araceli Garrido:** Visualization (equal); Writing‐review & editing (supporting). **Manuel Rendón‐Martos:** Visualization (equal); Writing‐review & editing (supporting). **Arnaud Béchet:** Funding acquisition (equal); Methodology (equal); Resources (equal); Visualization (equal); Writing‐review & editing (equal). **Juan A. Amat:** Conceptualization (lead); Data curation (equal); Formal analysis (equal); Funding acquisition (equal); Investigation (equal); Methodology (equal); Project administration (lead); Resources (equal); Software (equal); Supervision (lead); Validation (equal); Visualization (equal); Writing‐original draft (equal); Writing‐review & editing (equal).

## ETHICAL APPROVAL

Sample collection was authorized by the Office National de la Chasse et de la Faune Sauvage (now Office Français de la Biodiversité).

## Supporting information

Appendix S1

Appendix S2

## Data Availability

The data used in this study are presented as [Link], [Link]: https://doi.org/10.5061/dryad.bk3j9kdcr.
